# A retrospective analysis comparing metagenomic next-generation sequencing with conventional microbiology testing for the identification of pathogens in patients with severe infections

**DOI:** 10.3389/fcimb.2025.1530486

**Published:** 2025-04-08

**Authors:** Fei Hou, Yanting Qiao, Yuanyuan Qiao, Ya Shi, Mingrui Chen, Min Kong, Xiaohang Hu, Liqing Jiang, Xiaowei Liu

**Affiliations:** ^1^ Medical Laboratory of Jining Medical University, Jining Medical University, Jining, Shandong, China; ^2^ Department of Clinical Laboratory, The Affiliated Hospital of Jining Medical University, Jining, Shandong, China; ^3^ Department of Critical Care Medicine, The Affiliated Hospital of Jining Medical University, Jining, Shandong, China

**Keywords:** metagenomic next-generation sequencing, detection of pathogens, severe infections, bronchial alveolar lavage fluid, blood, conventional microbiological testing

## Abstract

**Introduction:**

The application value of metagenomic next-generation sequencing (mNGS) in detecting pathogenic bacteria was evaluated to promote the rational and accurate use of antibiotics. A total of 180 patients with severe infections were included in this study.

**Methods:**

Based on their different symptoms, bronchoalveolar lavage fluid (BALF) or blood samples were collected for conventional microbiological testing (CMT) and mNGS.

**Results:**

The results indicated that the etiological diagnosis rate of mNGS (78.89%) was significantly higher than that of CMT (20%) (p<0.001). Notably, mNGS exhibited greater sensitivity towards rare pathogens such as Chlamydia pneumoniae, Mycobacterium tuberculosis complex, and Legionella pneumophila, which were undetectable by CMT. Additionally, 64 cases underwent blood culture, BALF culture, and mNGS testing. Analysis revealed that the positive rate of blood culture (3.1%) was lower than that of BALF (25%), and the positive rate of CMT from both types was significantly lower than that of mNGS (89.1%) (p<0.001). In this study, 168 mNGS results were accepted, and 116 patients had their antibiotic therapy adjustment based on mNGS. Paired analysis indicated that white blood cell count (WBC), procalcitonin (PCT), C-reactive protein (CRP), and neutrophil (NEU) percentage provided valuable therapeutic guidance. The survival rate of patients was 55.36%, influenced by patient physical condition and age.

**Discussion:**

Our data indicated that mNGS had significant auxiliary value in the clinical diagnosis and treatment for critically ill patients, especially for those with negative CMT results and clinically undefined infections. mNGS could broaden the detection scope, especially for special pathogens, and improve the detection rate, providing powerful assistance for early clinical diagnosis and treatment.

## 1 Introduction

The most common pathogens causing infections include bacteria, fungi, viruses, and parasites, often manifested as fever, cough, dyspnea, etc. If not treated promptly, the disease might progress to complications such as multi-organ failure, septic shock, and sepsis. Despite continuous optimization of antibiotic used in clinical practice, the incidence and mortality rates of severe infections remained high (Papathanakos et al., 2023). This was particularly challenging in elderly patients with long-standing underlying conditions such as hypertension, diabetes, chronic kidney disease, chronic heart failure, and chronic obstructive pulmonary disease, which complicated the treatment process (Kahn et al., 2015; Di Pasquale et al., 2019; Zinter et al., 2019; Ohbe et al., 2021; Arvaniti et al., 2022). However, due to the limitations of past medical technology, patients’ conditions often progressed irreversibly before diagnostic results were available. For patients with severe infections, effective diagnosis and prognosis assessment would significantly aid doctors assess the severity of the patient’s condition, promptly adjust treatment plans, and rationally use targeted antibiotics and other supportive therapies to reduce drug resistance, as well as the mortality rates (Messacar et al., 2017; Calabretta et al., 2024).

Currently, methods for detecting pathogens had become increasingly sophisticated, including pathogen culture, serum-specific antigen-antibody detection, smear staining microscopy, and polymerase chain reaction (PCR) technology for specific pathogens. However, these conventional microbiological testing (CMT) had limitations such as long culture cycles, low detection sensitivity or specificity, and detection gaps for some rare pathogens (Wu et al., 2021; Yang et al., 2022), which could prolong the disease duration. Metagenomic next-generation sequencing (mNGS), as a novel pathogen detection method, was gradually being applied in infectious disease research, especially in areas where CMT had limitations. The emergence of this technology had greatly improved diagnostic efficiency and accuracy (Han et al., 2019). Through high-throughput sequencing of genetic fragments in samples, mNGS could obtain the genetic information of pathogens, offering advantages of rapidness, high sensitivity, broad coverage, and unbiasedness (Diao et al., 2022; Sun et al., 2022). The advantages of this technology were particularly evident in rare or emerging pathogens (Li et al., 2021; Kong et al., 2022; Liu et al., 2022), making it a high-throughput method for comprehensive analysis of the microbiome in clinical samples. Compared with CMT, mNGS had higher sensitivity in pathogen identification and was less affected by antibiotics (Miao et al., 2018). Consequently, it possessed the ability to detect infections, especially in patients experiencing acute onset. Early intervention with this treatment not only facilitated effective disease management but also enhanced the prognosis for patients.

In this study, we recruited a total of 180 patients with severe infections for a retrospective analysis. bronchoalveolar lavage fluid (BALF) and peripheral blood samples were collected for mNGS and CMT. Finally, the diagnostic performance of mNGS was evaluated by comparing with CMT.

## 2 Materials and methods

### 2.1 General information

Clinical data of patients admitted to the department of critical care medicine, Affiliated Hospital of Jining Medical university from June 2021 to May 2023 were collected, including gender, age, underlying medical history, clinical manifestations, length of hospital stay, routine examinations, and other basic information. Inclusion criteria for samples: 1. manifestations of pathogen invasion: clinical manifestations of infection such as fever, chills, cough and sputum; 2. manifestations of respiratory failure and dyspnea: parched lips, hypoxia, etc.; 3. varying degrees of organ damage; 4. the diagnosis of infection or co-infection was considered based on clinical manifestations, laboratory examination, imageological diagnosis and the clinical response to antibacterial drugs; 5. age of not less than 14 years; 6. patients who agreed to participate in this research and signed the informed consent. Exclusion criteria: 1. Pathogens determined using only one method; 2. Refusing to cooperate with the study; 3. the BALF/blood submitted for examination cannot meet the detection requirements (sample size ≥5mL), such as sample contamination. The study was approved by the Ethics Committee of our hospital (2023-06-C031).

### 2.2 Sample collection

#### 2.2.1 BALF collection

The targeted lung segment for lavage was determined based on clinical indications and imaging examinations. Subsequently, 2% lidocaine was administered to achieve mucosal surface anesthesia of the airway. A fiberoptic bronchoscope was then introduced into the designated lung segment, followed by the instillation of 25-50 mL of normal saline to perform bronchial lavage. After lavage, the recovered fluid was aspirated using negative pressure and transferred into a sterile container. Upon completion, the samples were then sent to the Laboratory Medicine for testing.

#### 2.2.2 Collection of peripheral blood bamples

2 mL of peripheral blood was collected and sent to the Laboratory Medicine for testing. All tests were conducted by the Laboratory Medicine of Affiliated Hospital of Jining Medical university.

### 2.3 Bacterial culture

#### 2.3.1 Blood culture

Thermo Scientific culture bottles were used for routine blood culture, with aerobic and anaerobic culture respectively. According to the bacterial and fungi culture procedure of the Laboratory of the Affiliated Hospital of Jining Medical university, routine separation media were used, including blood AGAR, chocolate AGAR and China Blue AGAR. blood AGAR and chocolate AGAR plates were cultured at 35°C with 8%CO2 for 120h. For special strains, the culture time was relatively extended. During this period, when there was a positive report, we would carry out the strains and transfer, MALDI-TOF-MS or identification cards were used for colony identification.

#### 2.3.2 BALF culture

According to the bacterial and fungi culture procedure of the Laboratory of the Affiliated Hospital of Jining Medical university, 10ul BALF samples were taken and planted on blood AGAR, Chinese blue AGAR and chocolate AGAR plates. The plates were cultured at 35~37°C with 5%~10% CO2 for 24~48h. The blood plates and chocolate plates were preferably cultured for 48h. For some special strains, the culture time was relatively extended. During this period, when there was a positive report, we would do the purification, and then the strains were identified by MALDI-TOF-MS or VITEK finally.

### 2.4 mNGS testing

#### 2.4.1 Pretreatment of BALF samples

The obtained BALF samples were sterilized at 65°C for 30 minutes. 480 μL of the sample was mixed with 7.2 μL of lysozyme and placed in a 30°C metal bath for 10 minutes. The sample was then transferred to a screw-cap tube containing 250 μL of 0.5 mm glass beads and placed in a shaker for maximum speed shaking for 20 minutes. After shaking, the sample was centrifuged at 8000 rpm for 30 seconds, and 300 μL of supernatant fluid was separated into a new 1.5 mL centrifuge tube for future use.

#### 2.4.2 DNA extraction from BALF/Blood samples

DNA was extracted according to the instructions of the DNA Purification Kit (Huada Biotechnology Co., Ltd., China). The DNA concentration was measured using the Qubit dsDNA HS Assay Kit 4.0.

#### 2.4.3 Preparation and sequencing of DNA libraries

DNA libraries were prepared using the PMseq™ High-throughput DNA Detection Kit (Huada Biotechnology Co., Ltd, China). After enzymatic digestion, end repair, adapter ligation, and PCR amplification of the DNA libraries, the DNA concentration of the sample was measured using the Qubit dsDNA HS Assay Kit 4.0. Eligible libraries were pooled into 0.2 mL PCR tubes. DNA nanospheres were prepared using the MiSeq™ Dx Kit (Huada Biotechnology Co., Ltd, China), and the samples were sequenced using the Huada BGI platform (Jeon et al., 2014).

#### 2.4.4 Bioinformatics analysis

Raw sequencing data was analyzed by a bioinformatic pipeline developed by BGI (Cai et al., 2020). The raw sequencing data was filtered to remove adapter sequences, low-quality sequences, short read lengths, and human sequences. The obtained non-human sequencing data was compared with a microbial genome database (http://ftp.ncbi.nlm.nih.gov/genomes/), which included 11910 bacteria, 7103 viruses, 1046 fungi, and 305 parasites associated with human diseases (Geng et al., 2021; Liang et al., 2022). High-quality sequencing data were first generated by removing short (length<35bp) and adapter-contained and high-N-base ratio and low-quality reads. Secondly, those reads from host cell were filtered by mapping the high-quality reads to human reference genome (hg38) use Burrows-Wheeler Aligner (Li and Durbin, 2009) and Kraken2 (Wood and Salzberg, 2014). Thirdly, non-host reads were mapped to plasmid database for plasmid removal. Then prinseq-lite tools (Schmieder and Edwards, 2011) was performed to remove low-complexity reads. The remaining sequencing data were finally aligned to the self-build PMseq database (PMDB, version 6.1.0) which refer to NCBI (https://www.ncbi.nlm.nih.gov/), FDA-ARGOS (https://www.ncbi.nlm.nih.gov/bioproject/231221) (Sichtig et al., 2019), DDBJ (https://www.ddbj.nig.ac.jp/index-e.html) (Fukuda et al., 2021) and EuPathDB (http://eupathdb.org) (Amos et al., 2022). For each batch of experiments, we established negative control (NC) and positive control (PC), using the same wet lab work flow and bioinformatics analysis as the clinical samples. Base on the alignment results of PMDB-v6, we calculated strict unique mapped reads number (smrn) for each species. The candidate positive criteria of mNGS were as follows: smrn was at least 3 for bacteria and fungi, at least 1 for virus and at least 100 for parasite, and the species were absent in NC or the smrn of clinic sample were 3-fold greater than NC when the smrn of NC were greater than 50 or the smrn of clinic sample were 5-fold greater than NC when the smrn of NC were less than or equal 50. Human colonization as well as environmental and reagent consumables common microorganisms such as Intestinimonas butyriciproducens (Bui et al., 2016), Acinetobacter pittii (Nemec et al., 2011), Acinetobacter guillouiae (Adler et al., 2012) and Xanthomonas campestris (Hodgetts et al., 2015) were excluded from candidate positive results. Subsequently, the genus level was sorted based on the smrn, and the top species from each genus level was selected as the final positive results. Most importantly, two or more physicians referred to the contents of the sequencing report, such as the number of unique reads, relative abundance, sequencing sample information, microbial genome size, etc., to comprehensively query the pathogenicity of microorganisms, combined with the clinical characteristics and examination results of patients, and compared the results of mNGS and CMTs with the final clinical diagnosis.

### 2.5 Statistical analysis

SPSS 20 statistical software was used for data analysis. Pairwise chi-square tests were conducted to compare the two different detection methods. *p* < 0.05 was considered statistically significant.

## 3 Results

**Figure 1:**
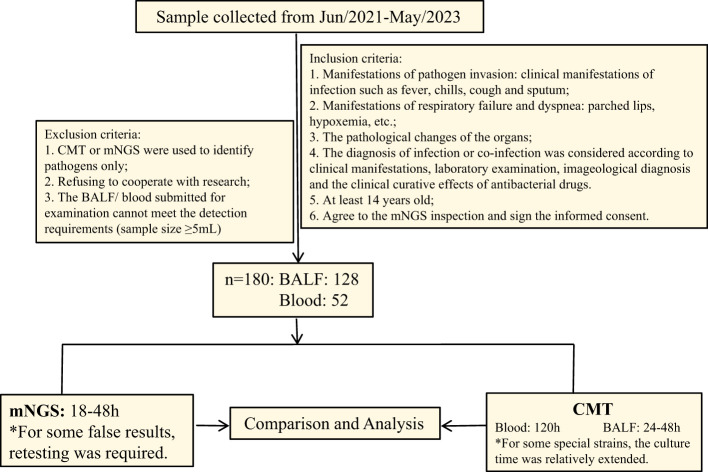
Flow diagram.

Based on **Figure 1**, we screened a total of 180 patients with severe infections, with an average age of 60.25 years. There were 124 male patients with an average age of 62.48 years and 56 female patients with an average age of 55.3 years. Pre-admission symptoms included fever in 65 patients, cough in 68 patients, expectoration in 51 patients, and dyspnea in 46 patients. There were 44 patients healthy before and 136 patients with underlying diseases. Among those with underlying diseases, 56 had hypertension, 29 had diabetes, 31 had cerebrovascular diseases, and 25 had lung diseases. The basic information was shown in **Table 1**.

**Table 1:** Summary of patients’ characteristics.

Feature	Total	Outcomes (n)			Three-month survival rate (n)	Chi-square test	
		Improvement	Critical condition	Death		χ2	*p* value
Male	124	63 (50.81%)	45 (36.29%)	16 (12.90%)	63 (50.81%)	4.314	0.116
Female	56	37 (66.07%)	12 (21.43%)	7 (12.50%)	36 (64.29%)		
Groups							
Past medical history	136	68 (50.0%)	49 (36.03%)	19 (13.97%)	67 (49.26%)	7.054	0.029
Be well before	44	32 (72.73%)	8 (18.18%)	4 (9.09%)	32 (72.73%)		
Age cohorts							
‗65	89	36 (40.45%)	38 (42.70%)	15 (16.85%)	35 (39.33%)	16.284	<0.001
<65	91	64 (70.33%)	19 (20.88%)	8 (8.79%)	64 (70.33%)		
Disease classification							
Diabetes	29	10 (34.48%)	14 (48.28%)	5 (17.24%)	10 (34.48%)	16.77	<0.001
Hypertension	56	19 (33.93%)	27 (48.21%)	10(17.86%)	18 (32.14%)	46.47	<0.001
Nephropathy	9	4 (44.44%)	4 (44.44%)	1 (11.11%)	4 (44.44%)	6.568	0.0104
Hepatic disease	8	3 (37.5%)	4 (50.0%)	1 (12.5%)	3 (37.5%)	3.2	0.0736
Hematological Disease	10	5 (50.0%)	5 (50.0%)	0 (0)	5 (50.0%)	7.302	0.0069
Heart Disease	46	23 (50.0%)	15 (32.61%)	8 (17.39%)	22 (47.83%)	17.75	<0.001
Cerebral disease	31	8 (25.81%)	16 (51.61%)	7 (22.58%)	8 (25.81%)	17.18	<0.001
Pulmonary disease	25	17 (68.0%)	7 (28.0%)	1 (4.0%)	17 (68.0%)	9.829	0.0017

### 3.1 Comparison of mNGS and CMT

All 180 samples in this study were tested for pathogens using both methods, including 128 BALF samples and 52 blood samples. mNGS identified 149 positive reports, including 113 (113/128, 88.28%) in BALF and 36 (36/52, 69.23%) in peripheral blood. CMT identified 36 positive reports, including 28 cases (28/128, 21.88%) in BALF and 8 cases (8/52, 15.38%) in blood, the positive rate of mNGS (82.78%, 149/180) was higher than that of CMT (20%, 36/180) (p<0.001). A total of 67 types of bacteria, 17 types of fungi, and 12 types of viruses were detected by mNGS in BALF. The pathogen spectrum showed that the most common Gram-positive bacteria were Streptococcus pneumoniae (24/128, 18.75%), Streptococcus constellatus (13/128, 10.16%), and Enterococcus faecalis (6/128, 4.69%); the main Gram-negative bacteria were Haemophilus influenzae (17/128, 13.28%), Pseudomonas aeruginosa (13/128, 10.16%), and Acinetobacter baumannii (6/128, 4.69%); the most common fungi were Candida albicans (13/128, 10.16%), Aspergillus fumigatus (9/128, 7.03%), and Pneumocystis jirovecii (4/128, 3.13%); and the most common viruses were Human herpesvirus 4 (EBV) (21/128, 16.41%), Human herpesvirus 1 (HSV-1) (8/128, 6.25%), Human herpesvirus 7 (HHV-7) (8/128, 6.25%), and Torque teno virus (TTV) (8/128, 6.28%). A total of 17 types of bacteria, 8 types of fungi, and 13 types of viruses were detected by mNGS In blood samples. The most common Gram-positive bacteria were Enterococcus faecium (3/52, 5.77%) and Staphylococcus aureus (3/52, 5.77%); the main Gram-negative bacteria were Klebsiella pneumoniae (7/52, 13.46%), Acinetobacter baumannii (4/52, 7.69%), Escherichia coli (3/52, 5.77%), and Pseudomonas aeruginosa (3/52, 5.77%); the most common fungi were Aspergillus fumigatus (4/52, 7.69%); and the most common viruses were Human herpesvirus 5 (CMV) (11/52, 21.15%) and EBV (7/52, 13.46%). Compared with mNGS, CMT of blood detected 6 types of bacteria, mainly Staphylococcus aureus (3/52, 5.77%); CMT of BALF detected 10 types of bacteria and 3 types of fungi, with the main bacteria being Klebsiella pneumoniae (5/128, 3.91%) and the main fungi being Candida albicans (5/128, 3.91%) and Aspergillus fumigatus (4/128, 3.13%) (**Supplementary Table**). In addition to the comparisons of the positive results mentioned above, we also had new discoveries. mNGS detected Chlamydia psittaci (2 cases), Mycobacterium tuberculosis complex (5 cases), and Legionella pneumophila (2 cases), while these rare pathogens were undetected by CMT. Moreover, the mNGS were adopted clinically and guided the medication to adjust in time, ultimately resulting in improvements in the patients’ conditions.

**Figure 2:**
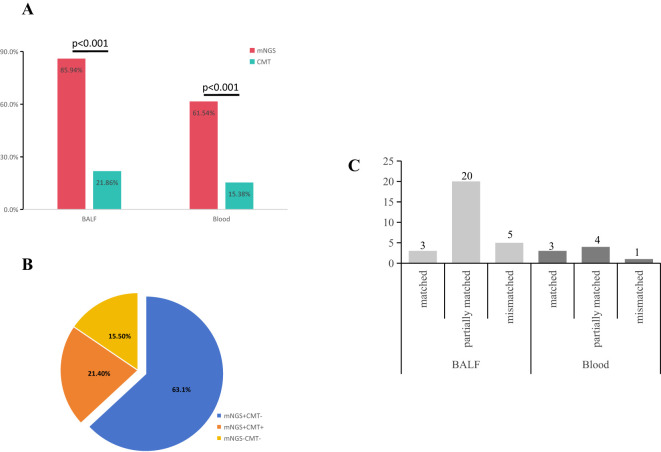
Concordance of samples between mNGS and CMTs. **(A)** The positive rates of pathogen identification between mNGS and CMT in BALF (85.94%, 110/128 vs 21.86%, 28/128) (p<0.001) and blood (61.54%, 32/52 vs 15.38%, 8/52) (p<0.001). **(B)** Concordance of diagnosis between mNGS and CMT. 36 cases (21.4%, 36/168) were both positive, 26 cases (15.5%, 26/168) were negative for both methods, and 106 cases (63.1%, 106/168) were positive by mNGS only. **(C)** There were 3 cases (10.7%) were matched, 18 cases (64.3%) were partially matched, and 7 cases (25%) were completely mismatched in BALF. 3 cases (33.3%) were matched, 5 cases (55.6%) were partially matched, and 1 case (11.1%) was mismatched in Blood.

To further evaluate the consistency between mNGS and CMT results, we conducted a comparative analysis of these two methods. Based on the test outcomes, clinical indications, and various auxiliary examinations, 7 cases of mNGS were identified as false positives, while the remaining 142 patients received clear etiological diagnoses. The diagnosis rate of mNGS (78.89%, 142/180) was significantly higher than that of CMT (20%, 36/180) (p<0.001) (**Figure 2A**). Among the 168 severe cases, 21.4% (36/168) tested positive by both mNGS and CMT, 63.1% (106/168) were positive only by mNGS, none were positive only by CMT, and 15.5% (26/168) were negative by both methods (**Figure 2B**). In samples that tested positive by both methods, 3 cases of plasma samples (3/52, 5.77%) showed complete concordance, 4 cases (4/52, 7.69%) exhibited partial concordance, and 1 case (1/52, 1.92%) had no matching results. For BALF samples, 3 patients (3/128, 2.34%) had completely matched, 20 patients (20/128, 15.63%) had partially matched, and 5 patients (5/128, 3.91%) had completely unmatched (**Figure 2C**). This comparison demonstrated that mNGS had a significantly higher etiological diagnosis rate than CMT, which could effectively guide the clinical adjustment of antibiotics.

Furthermore, among the 128 enrolled BALF samples, 64 samples were also subjected to blood culture simultaneously. The results showed that the blood culture had a positive rate of 3.1% (2/64), while the BALF culture had a positive rate of 25.0% (16/64), and mNGS had a positive rate of 89.1% (57/64). There was a statistically significant difference in the overall mean among the three groups (χ² = 107.257, *p* < 0.001). The sensitivity of BALF culture was higher than that of blood culture (*p* < 0.05), and there were statistically significant differences in sensitivity between mNGS and both blood culture and BALF culture (*p* < 0.05) (**Table 2**).

**Table 2:** A comparative analysis of the sensitivity of three distinct methods for pathogenic bacteria.

Groups		Total (n)	Negative (n,%)	Positive (n,%)	Chi-square test	
					χ²	*p* value
CMT	Blood	64	62, 96.9	2, 3.1	107.257	<0.001
	BALF	64	48, 75.0	16, 25.0 *		
mNGS		64	7, 10.9	57, 89.1 #*		

*Compared with blood, there was statistical significance; #Compared with BALF, there was statistical significance.

### 3.2 The result of mNGS were concordanced with clinical findings

**Figure 3:**
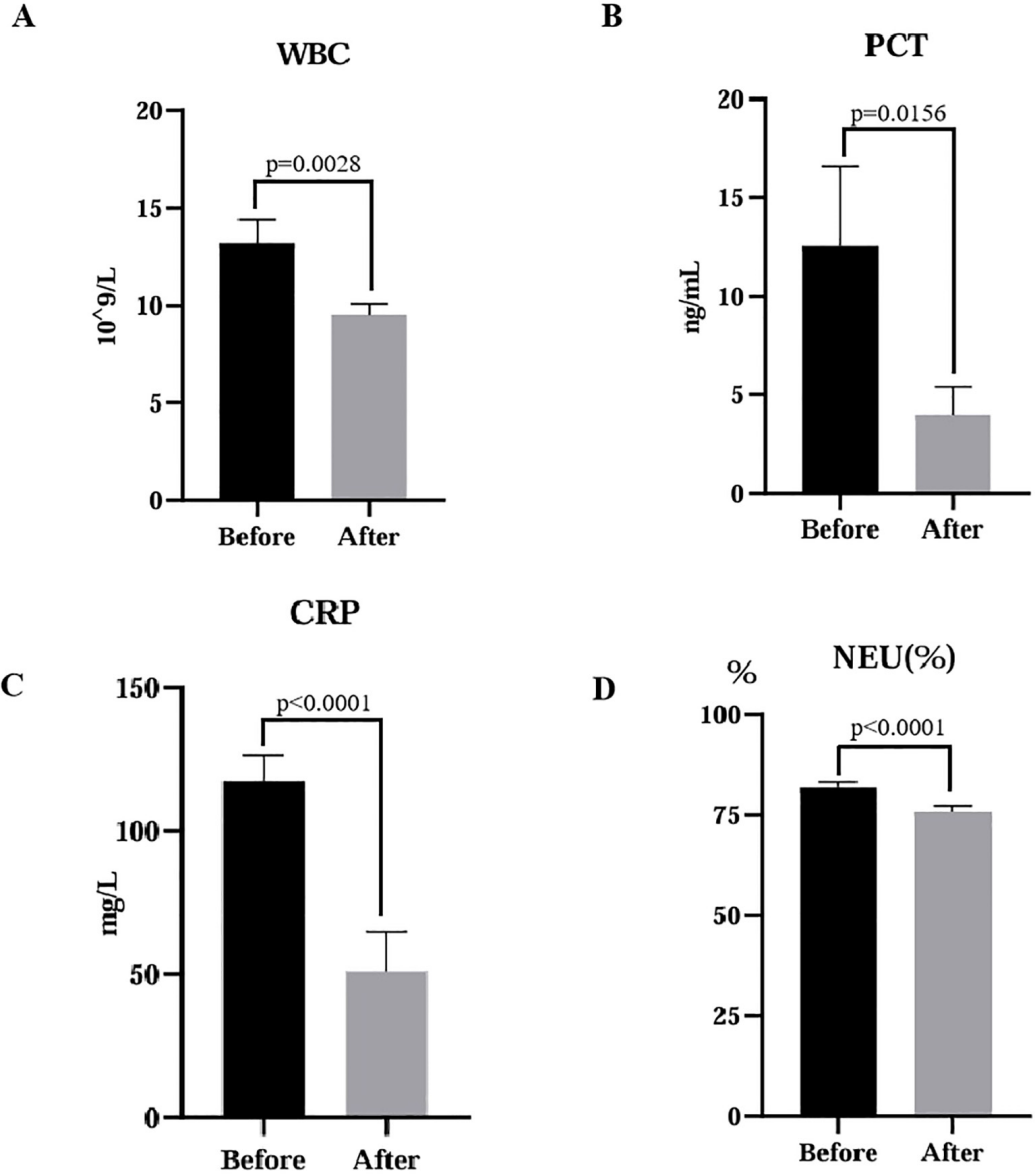
Comparative analysis of laboratory indicators before and after treatment. Statistical significance was determined using a paired t-test. **(A)** WBC, white blood cell, *p*=0.0028; **(B)** PCT, procalcitonin, *p*=0.0156; **(C)** CRP, C-reactive protein, *p*<0.0001; **(D)** NEU, neutrophils, *p*<0.0001.

In this retrospective analysis, a total of 168 mNGS results were adopted. Treatment adjustments were made for 116 patients based on the mNGS, and we conducted paired analysis on biochemical indicators collected from these patients. The study demonstrated that there were significant alterations in the level of white blood cell (WBC), C-reactive protein (CRP), neutrophil (NEU) percentage, and procalcitonin (PCT) following treatment. The changes in WBC (*p*=0.0028), PCT (*p*=0.0156), CRP (*p*<0.0001), and NEU% (*p*<0.0001) were statistically significant (**Figure 3**), indicating their potential as indicators for monitoring therapeutic response in infectious diseases. Although the biochemical indicators of patients suggested initial improvement in treatment efficacy after adopting the mNGS results, a part of patients still experienced worsening conditions or even death due to rapid disease progression and other complications.

### 3.3 Three month survival rate

**Figure 4:**
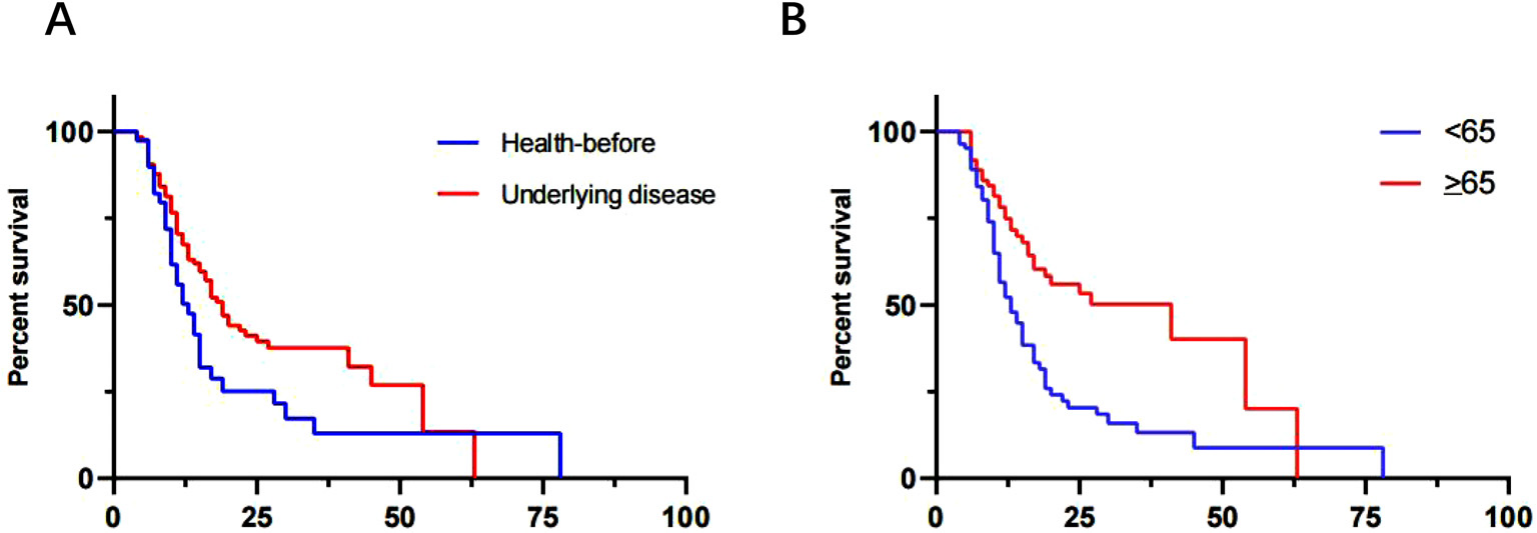
Three-month survival rate analysis. **(A)** Patients were well previously vs Patients underlying medical conditions (*p*=0.0278). **(B)** ≥65 vs <65 (*p*=0.0002).

To evaluate the clinical significance of mNGS, a cohort of 168 patients was monitored, and their 90-day survival rates were assessed. The overall 90-day survival rate was 55.36% (93/168). Notably, previously healthy patients exhibited a significantly higher survival rate of 75.61% (31/41) compared to patients with underlying disease, who had a survival rate of 48.82% (62/127), with this difference being statistically significant (χ²=4.84, p=0.0278) (**Figure 4A**). Additionally, patients younger than 65 years old demonstrated a markedly higher 90-day survival rate of 70.11% (61/87) compared to those aged 65 or older, whose survival rate was 39.51% (32/81), a difference that was also statistically significant (χ²=14.0, p=0.0002) (**Figure 4B**). The sensitivity and rapid turnaround time of mNGS results might facilitate timely adjustments in treatment strategies and improve patient survival.

## 4 Discussion

Patients with severe infections were typically characterized by rapid disease progression and elevated mortality rates. This was particularly evident among elderly patients, who exhibited higher hospitalization and mortality rates (Zhang et al., 2022), especially those with underlying diseases such as diabetes, hypertension, coronary heart disease, and others (MaChado-Curbelo et al., 2022). As age increases, these individuals became more susceptible to complications during the course of illness. In our study, 75.56% of the included patients had underlying diseases, which significantly contributed to poor prognosis in certain cases. Hence, the rapid identification of pathogenic bacteria in affected was crucial for effective treatment timely. Historically, CMT served as the primary diagnostic method in most hospitals. However, it demonstrated a low incidence of positive infections, and required cultures that could take anywhere from 2-14 days or even up to 6 weeks for results. Moreover, traditional cultures often lack sensitivity-particularly concerning slow-growing or fastidious microorganisms and fungi-which presented considerable limitations for patients experiencing acute illness onset. mNGS had alleviated these constraints by providing a rapid and sensitive auxiliary diagnostic tool that could identify all known microorganisms present in clinical specimens within a short turnaround time (48 hours), utilizing high-throughput sequencing coupled with automated bio-informatics analysis. Currently, mNGS had been employed for pathogen detection across various sample types including cerebrospinal fluid (Wilson et al., 2014; Guan et al., 2016; Su et al., 2024), blood (Grumaz et al., 2020), urine (Mouraviev and McDonald, 2018; Gu et al., 2021), pleural effusion (Zhu et al., 2022; Xu et al., 2023), BALF (Miao et al., 2018; Yang et al., 2022; Hu et al., 2024) and lesion tissues (Huang et al., 2020; Kong et al., 2022). It effectively enhanced analytical sensitivity for microorganism identification and mixed infection diagnosis, while being less influenced by medications-a promising advantage in severely ill patients.

In this retrospective analysis, we found that the positive rate of etiological diagnosis for mNGS in BALF (85.94%) was significantly greater than that observed with CMT detection (21.88%). Studies had shown that the positive rate of CMT in blood was below 20%, compounded by longer culture time required for results (Chen et al., 2021; Sun et al., 2022). Our data further indicated that CMT positive rate for etiological diagnosis in blood samples (8/52, 15.38%) was markedly lower than that achieved via mNGS detection (32/52, 61.54%). The high negative rate associated with CMT might be attributed to prior anti-infective treatments received by patients before testing, thereby diminishing its sensitivity towards pathogens detection to some extent. Furthermore, negative mNGS reports also held clinical significance since they assisted clinicians in ruling out infections while offering diagnostic insights regarding tumors (Liu et al., 2021). Nonetheless, we acknowledged potential unreliability issues surrounding, mNGS results: based on clinical feedback from our cohort comprising 180 samples, 12 false results were identified, including seven false positives and five false negatives. Possible reasons behind false positives might include: contamination during sampling; cross-contamination throughout experimental process; laboratory background bacteria; or misinterpretation of outcomes. When clinicians expressed concerns over these positive findings, clinicians would re-evaluate the suspected false-positive samples considering actual circumstances along with patients’ symptoms. False negatives detected via mNGS necessitate reassessment against clinical data or required re-sampling efforts (Ma et al., 2022; Qian et al., 2022). Additionally, the type of clinical specimen utilized might have contributed to negative result occurrences. In our study, 64 patients underwent simultaneous applications of three diagnostic methods to detect pathogens. The results indicated that the positive rate of CMT in BALF (25.0%) was significantly higher compared to blood culture (3.1%). However, the sensitivity of CMT for pathogen detection was markedly lower than that of mNGS (89.1%). This underscored the necessity for clinicians to comprehensively evaluate the patients’ clinical symptoms, infection site, medical history, and lifestyle when selecting appropriate sample types for testing, as such considerations would enhance accurate clinical diagnosis (Xu et al., 2019) and potentially reduce healthcare costs concurrently. In addition, mNGS had more advantages than CMT in the positive detection rate of fungi. However, when dealing with colonizing fungi that are non-pathogenic bacteria, clinicians must consider a multiple of factors, including clinical manifestations, laboratory test results, imaging findings, routine diagnostic reports, and the outcomes of previous anti-infective therapies, to facilitate the differentiation between fungal colonization and infection.

Fortunately, mNGS demonstrated a considerably higher detection rate for certain rare pathogens compared to CMT, which held substantial clinical significance. A comparative analysis between two methods revealed that Mycobacterium tuberculosis complex, Chlamydia psittaci, and Legionella pneumophila were all negative via CMT but positive through mNGS; consequently, clinicians utilized mNGS results to inform timely adjustments in treatment regimens. Mycobacterium tuberculosis was an intracellular bacterium notoriously difficult to culture; thus, short-term CMT results were frequently negative while Computed Tomography (CT) exhibited relatively low sensitivity—factors that might contribute to delayed patient treatment (Jin et al., 2022; Li et al., 2024). Moreover, considering its infectious nature, misdiagnosis or delayed diagnosis would cause significant distress not only to patients but also their close contacts. Therefore, achieving timely diagnosis posed a considerable challenge for clinicians. In mNGS assessments where ≥1 read indicated Mycobacterium tuberculosis complex presence suggested notable clinical relevance necessitating integration with other diagnostic tests.

Furthermore, Legionella pneumophila represented an atypical pathogen capable of inducing fatal acute respiratory infections through inflammatory responses and parenchymal lung lesions (Li et al., 2023)—a condition often indistinguishable from other pneumonia types. The sensitivity of CMT towards Legionella was notably low and frequently yielded negative outcomes as well (Lei et al., 2022). Additionally, research had established that serum conversion typically occurred only three weeks post-infection in most cases of legionnaires’ disease—with over one-quarter never achieving serum conversion—thereby increasing the likelihood of delayed diagnoses (Cunha et al., 2016). Consequently, the advent of mNGS had substantially improved early diagnostic rates for Legionella infections. Patients infected with Chlamydia psittaci usually presented with a history indicative of poultry exposure; imaging studies could also reveal lesions aiding early diagnosis and evaluation of therapeutic efficacy (Wu et al., 2023). Nevertheless, the implementation of mNGS enhanced the diagnostic probability associated with Chlamydia psittaci pneumonia whereby prompt administration of suitable antibiotics could yield favorable prognoses (Fang et al., 2022). Thus, for certain rare bacterial infections often overlooked by CMT—which might lead to severe complications—it was imperative that clinicians maintain heightened clinical awareness. For patients who remained misdiagnosed via CMT, timely application of mNGS served as an auxiliary tool, facilitating rapid identification and management strategies against rare bacteria contributing positively toward expeditious treatment outcomes (Off, 2023).

In order to assess the efficacy of mNGS in critically ill patients, a three-month follow-up revealed that the survival rate was 55.0%. Among them, the survival rate of 168 patients with mNGS results adopted was 55.36%. The survival rate for patients with previous health conditions (75.61%) was much higher than that of patients with underlying disease history (48.82%), especially for some patients with cerebrovascular diseases, such as cerebral infarction, the survival rate was only 25.81%(8/31). The survival rate decreased as age increased, form 70.11% to 39.51%. Even with clinical interventions, patient prognosis remained vulnerable to multiple factors, such as age, health conditions, and manifestations of disease severity, all of which contributed to the low survival rates. Patients suffering from severe infections often had chronic underlying diseases, which weakened their resilience compared to healthy individuals. Furthermore, they were subject to prolonged medication regimens. It could have adverse effects on bodily functions, making them more susceptible to pathogen invasion and subsequent physical injury. As a result, antibiotic treatments often proved ineffective, and the prognoses for these patients were equally poor. In clinical practice involving severely ill patients lacking definitive microbiological diagnoses, empirical broad-spectrum antibiotics were typically administered initially to alleviate symptoms during early disease stages (Zhuang et al., 2017; Charalampous et al., 2019); if favorable responses were observed in patient’s condition, clinicians might continue this regimen, inadvertently leading to overuse or misuse of broad-spectrum antibiotics due to the lack of identification of the involved pathogens. Thus arising a significant challenge: transitioning from empirical treatment paradigms towards precision medicine aimed at enhancing patient outcomes—a necessity that must be addressed within clinical settings (Li et al., 2018). For patients with severe condition, mNGS could not only improve the diagnosis rate and shorten the diagnosis time, but also further improve the accuracy of clinical anti-infection treatment, which was not achieved by CMT, and then effectively improve the clinical status of patients (Wu et al., 2024).

Despite the application of mNGS in clinic, its practical implementation remained challenging owing to the absence of standardized guidelines, encompassing indications for mNGS testing, quality control measures, data analysis protocols, result interpretation and others (Calabretta et al., 2024; He et al., 2024). Moreover, the inherent complexity of mNGS posed significant challenges in accurately interpreting results, particularly in distinguishing colonization and infection, especially the opportunistic infections (Liu et al., 2023), background interference further complicated this distinction (Miller et al., 2019). Additionally, the high-cost limited its widespread adoption as a first-line diagnostic tool (Tan et al., 2022). However, our study focused on pathogen DNA sequencing might overlook RNA virus infections, leading to potential detection gaps (Fan et al., 2023; Liu, 2024).

In this retrospective analysis, our findings highlighted the advantages of mNGS. However, the limited scale of this study restricted its ability to fully capture the correlation between mNGS and clinical indicators. Study had showed that mNGS might significantly enhance diagnostic accuracy in our study but do not adequately represent its utility in other contexts, such as complex infections, febrile cases, or critically ill patients in different regions. Future studies could adopt comprehensive approaches, such as the method proposed by Katrina et al (Kalantar et al., 2022), we might detect RNA and DNA simultaneously, thereby minimizing detection gaps. In addition, The relatively small sample size coupled with incomplete clinical data had impacted data analysis results significantly. To further evaluate applicability value of mNGS among critically ill populations, additional participants were required, which would enhance overall analysis in turn. While findings indicated that mNGS demonstrated superior performance relative CMT regarding pathogen detection—it should not serve as an absolute substitute but rather function as an auxiliary diagnostic tool assisting clinicians thereby facilitating precision-based therapeutic approaches.

In conclusion, it was evident that severe infectious diseases presented considerable complexity alongside diagnostic challenges. The prolonged use of broad-spectrum antibiotics has been shown to be ineffective, and the increase of resistant bacterial strains posed significant risks to public health. In the department of clinical critical care, particularly for patients with negative CMT results and ambiguous infection diagnostics, mNGS could enhance detection capabilities notably, such as atypical pathogens, and improve identification rates, thereby offering crucial assistance in facilitating swift diagnosis and effective treatments, while reducing the risks associated with empirical antibiotic administration and poor mismanagement. Moreover, a part of critically ill subjects experienced mixed infections, clinicians could utilize the results formed by mNGS to adjust diagnostic approaches and treatment protocols, implementing targeted anti-infective therapies as necessary.

## Data Availability

We have upload the data to the CNGB Sequence Archive (CNSA) of China National GeneBank DataBase, accession number CNP0007066.
